# Construction of ceRNA network and key gene screening in cervical squamous intraepithelial lesions

**DOI:** 10.1097/MD.0000000000031928

**Published:** 2022-12-02

**Authors:** Ding Qi, Hongmei Li, Shuoqi Wang, Shimeng Wang, Rui Zheng, Ning Liu, Buwei Han, Li Liu

**Affiliations:** a Heilongjiang University of Traditional Chinese Medicine, Heilongjiang, China; b The 2nd Affiliated Hospital of Heilongjiang University of Traditional Chinese Medicine, Heilongjiang, China; c The 1st Affiliated Hospital of Heilongjiang University of Traditional Chinese Medicine, Heilongjiang, China.

**Keywords:** ceRNA, cervical intraepithelial neoplasia, MAPK signaling pathway, VEGFA

## Abstract

This study aimed to construct an endogenous competition network for cervical squamous intraepithelial lesions using differential gene screening.

**Methods:**

GSE149763 was used to screen differentially expressed long non-coding RNAs (lncRNAs) and mRNAs to predict correlated microRNAs (miRNAs). The correlated miRNAs and GSE105409 were used to screen differentially expressed miRNAs for differential co-expression analysis, and the co-expressed differentially expressed miRNAs were used to predict correlated mRNAs. Differentially expressed mRNAs, miRNAs, and lncRNAs were visualized, and differential gene screening, enrichment, and pathway analysis were performed.

**Results:**

The ceRNA network of cervical squamous intraepithelial was successfully established and a potential differentially expressed network was identified. The key genes were VEGFA and FOS, and the key pathway was the MAPK signaling pathway.

**Conclusions:**

The differential expression and potential effects of the lncRNA BACH1-IT1/miR-140-5p/VEGFA axis, key genes, VEGFA and FOS, and MAPK signaling in CIN were clarified, and the occurrence and potential effects of CIN were further clarified. The underlying molecular mechanism provides a certain degree of reference for subsequent treatments and experimental research.

## 1. Introduction

A cervical squamous intraepithelial lesion (SIL), previously known as cervical intraepithelial neoplasia (CIN), is the abnormal proliferation of squamous epithelial cells under the action of various factors. It is characterized by poor differentiation, disordered arrangement, abnormal nuclei, and increased mitosis. In later stages, accompanied by complex interactions between viral oncogenes and host cells, they can gradually evolve into invasive cervical cancer.^[1]^ The Affected population are more common among women aged 25 to 35 years old. Although most low-grade squamous intraepithelial lesions can spontaneously regress, low-grade or high-grade lesions that have not been systematically treated, the transformation from dysplasia to invasive cervical cancer may occur within a few years or 10 years, and about 10% of patients, under the action of certain driving factors, can complete this transformation within 1 year.^[2]^ Women are usually asymptomatic because of their insidious clinical manifestations and lack of specific symptoms. Occasionally, women only behave as vaginal discharge increases or contact bleeding occurs after sexual activity and gynecological examination. The naked eye can see that the shape of the cervix is normal, the surface is relatively smooth, a cervical erosion-like change is observed, and obvious lesions are usually invisible. With the continuous improvement in the disease, the accuracy of its diagnosis has gradually improved. Surgical treatment is an effective method for the treatment of high-grade lesions.^[3]^ Although the lesions can be removed, patients must bear the risks of anesthesia, postoperative bleeding, and postoperative cervical adhesions.^[4]^ At the same time, with an increase in the incidence rate, the affected group is gradually become younger. This treatment method will undoubtedly have many adverse effects on the long-term lives of patients. The mid-term miscarriage rate has increased, as have the risks of preterm birth,^[5]^ low birth weight, premature rupture of membranes, and perinatal mortality. Therefore, finding a new noninvasive method for the timely treatment of cervical squamous intraepithelial lesions is a preventive measure. Effective measures for invasive cervical cancer are of great significance in improving women’s health status and maintaining fertility.

**Figure 1.**
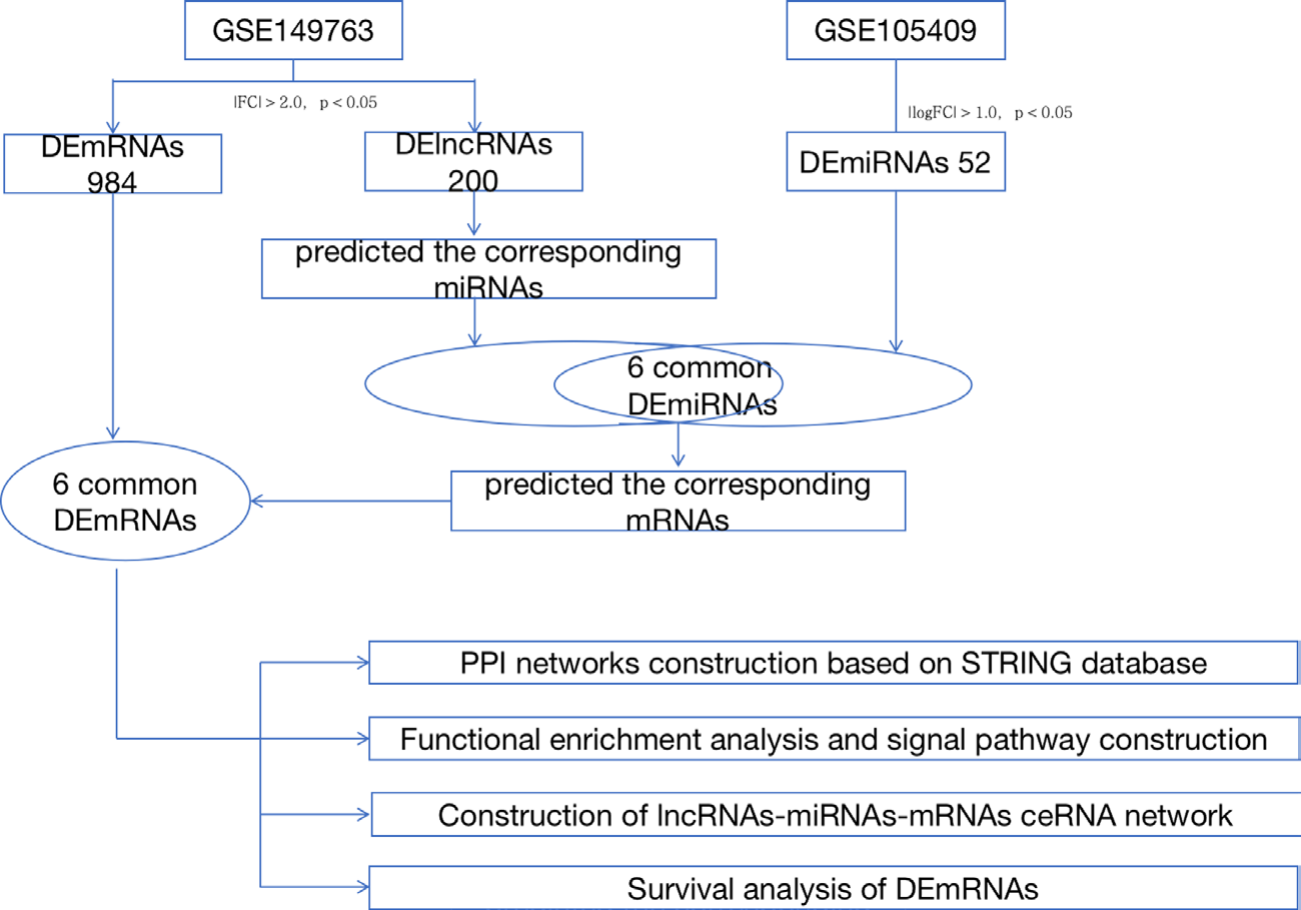
Flow diagram.

Innovative research on cervical squamous intraepithelial lesions has focused on clinical treatment methods and drug development.^[6]^ However, the specific mechanisms underlying cervical squamous intraepithelial lesions at the molecular level remain unclear. In recent years, owing to scientific and technological progress, the roles of lncRNAs (long non-coding RNAs), microRNAs (miRNAs), and mRNAs have been gradually explored, and their interaction mechanisms have gradually been clarified. Most of the expressed transcripts do not encode proteins, and transcripts with a length >200 nt are widely classified as lncRNAs, which are found in a variety of cells. It plays a role in biological processes (BP), including the cell cycle, metabolism, virus infection, and disease occurrence, and can affect diseases by interacting with DNA or binding to signal receptors.^[7]^ The length of miRNA is 21 to -22 nt, an miRNA can regulate multiple target genes at the same time, and plays a role in the post-transcriptional regulation of target genes.^[8]^ The length of mRNA is more than 100 to 150 nucleotides, and it mainly relies on in vitro enzymes to promote synthesis.^[9]^ The three can be constructed into a competitive endogenous RNA network (ceRNA), the main mechanism of which is the competition of shared miRNAs to regulate other RNA transcripts, given that each miRNA may compete with many transcripts for binding, and the competition of these transcripts is affected by their respective binding affinity to miRNAs. Generally speaking, the higher the affinity, the stronger the competitive ability.^[10]^ miRNAs are located at the center of ceRNA interactions, participate in the occurrence of various diseases, and have therapeutic potential. Post-transcriptional regulation is mediated by miRNAs that link the functions of coding and non-coding RNAs.^[11]^ In terms of cervical diseases, the molecular mechanism research is mostly cervical cancer, and there are few reports on the special research on cervical squamous intraepithelial lesions. Screening of key genes provides new insights for further research on this disease. The process is illustrated (Fig. 1).

## 2. Materials and methods

### 2.1. Data sources

Datasets that met prediction requirements were selected from the GEO database (https://www.ncbi.nlm.nih.gov/geo/). The detection samples must include normal and cervical squamous intraepithelial lesion tissues, respectively.^[12]^ At the same time, the detection method, sequencing technology and clinical case information are complete detection technology requires the use of the same method. Based on the relevant requirements, GSE149763 was selected to construct differential expression profiles of lncRNAs and mRNA between cervical squamous intraepithelial lesions and normal groups.^[13]^ GSE105409 dataset was used to verify the predicted miRNAs.^[14]^ GSE149763 used high-throughput sequencing as the detection method and included three patients with cervicitis, three with CIN III, and three with cervical cancer. GSE105409 detected 16 normal or cervicitis patients using an array analysis. Differential miRNA expression analysis was performed in 40 patients with CIN lesions and 30 patients with cervical cancer. We entered the differential expression lncRNAs transcript name into the Ensembl website (http://ensemblgenomes.org), converted it into a gene ID, and used it for subsequent visualization studies.

### 2.2. Screening of miRNAs

The GSE149763 dataset uses the online analysis tool GEO2R to analyze the stability of the data, download the original data, and use the limma package in R software to analyze the difference data.^[15]^ The expressed lncRNAs were screened to obtain differentially expressed lncRNAs (DELs) and their sequences,^[13]^ the miRNAs associated with DELs were predicted using lncRNA sequences and the miRcode database (http://www.mircode.org).^[16]^ This data include many non-coding genes, which can provide “whole transcriptome” human miRNA target prediction with reliable data information. The predicted miRNAs were intersected with the differentially expressed miRNAs in the GSE105409 dataset for further analysis.^[14]^ The GSE105409 dataset contains multiple cases for a comprehensive comparison of differentially expressed miRNAs in normal patients, cervical squamous intraepithelial lesions, and cervical cancer patients.

### 2.3. mRNA screening

Differentially expressed miRNAs (DEMs) were obtained using the miRDB (http://mirdb.org), Targetscan database (https://www.targetscan.org/vert_80/), and mirtarbase database (http://mirtarbase. cuhk.edu.cn/~miRTarBase/miRTarBase_2022/php/index.php) for correlated mRNA prediction,^[16]^ and the predicted results were co-expressed with the differentially expressed mRNAs screened in the GSE149763 dataset,^[13]^ and the intersection mRNA was screened out.

### 2.4. Construction of protein‐protein interactions (PPI) network

The differentially expressed genes (DEGs) were uploaded to the STRING database (https://string-db.org/cgi/input.pl) with the default conditions, the interaction network was obtained, and used Cytoscape version 3.9.0 to visualize all results,^[17]^ and the core genes and their regulatory relationships were displayed using the Cytohubba plug-in and network topology analysis functions.

### 2.5. Functional enrichment analysis

Functional enrichment analysis and pathway screening were performed using DAVID (https://david.ncifcrf.gov) and KEGG (Kyoto Encyclopedia of Genes and Genomes) database (https://www.kegg.jp).

### 2.6. Construction of ceRNA network

The screened DELs, DEMs, and DEGs were used to construct the interaction network using the Cytoscape software (version 3.9.0). Based on the degree of the link, ceRNA paths with high research value were screened and visualized.

### 2.7. Analysis of the prognosis of DEGs

Using the GEPIA website (http://gepia.cancer-pku.cn/detail.php), co-expressed DEGs were used for survival and prognosis analyses.

## 3. Results

### 3.1. RNA expression of cervical squamous intraepithelial lesions

**Figure 2.**
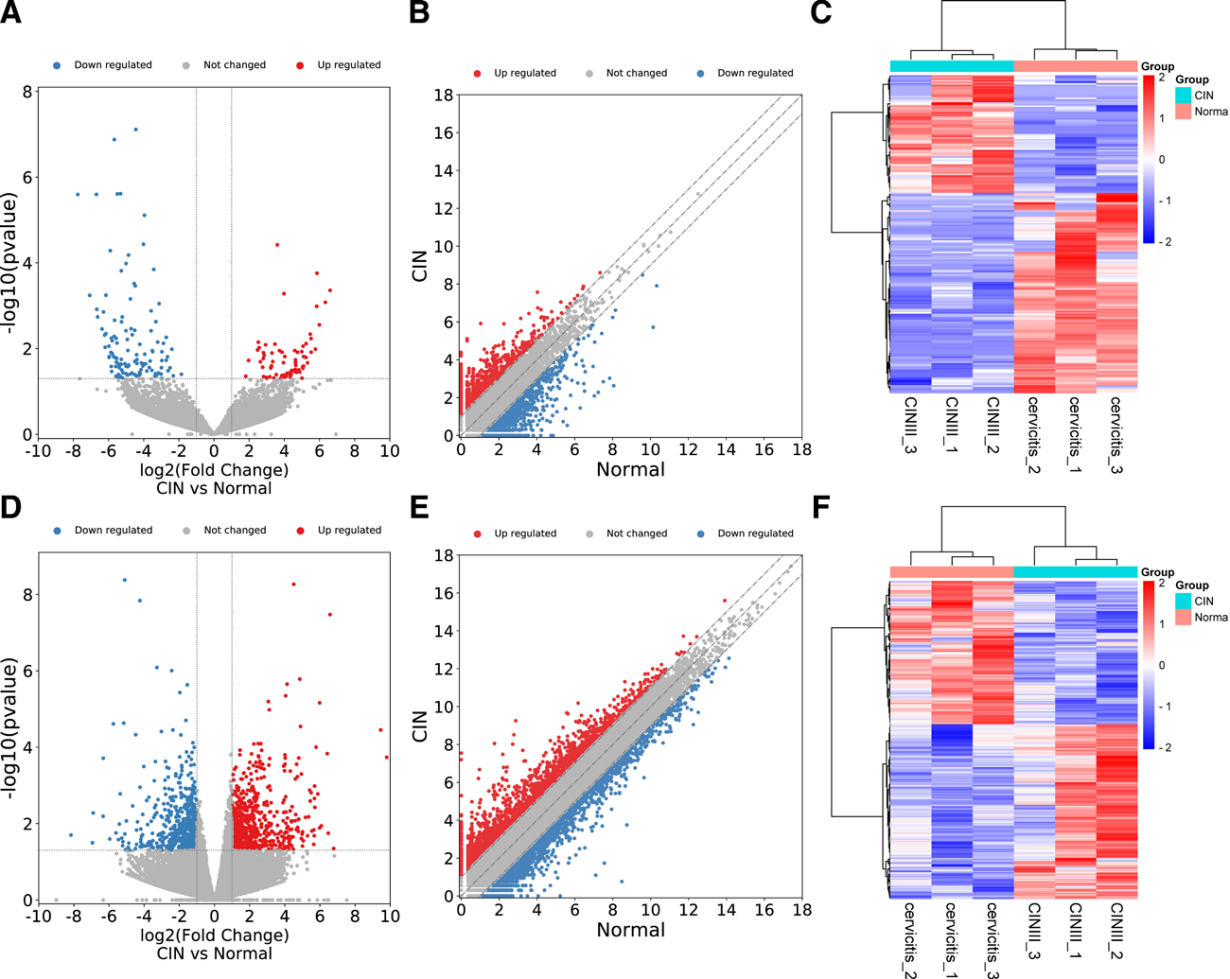
Identification of significant changes in RNAs expression in CIN. (A) Scatter plot, (B) volcano plots, and (C) heatmap with red for upregulation and dark blue for downregulation of lncRNAs in GSE149763. (D) Scatter plot, (E) volcano plots, and (F) heatmap with red for upregulation and dark blue for downregulation of mRNAs in GSE149763.

Using the GSE149763 dataset, the data transcript IDs were converted into gene IDs using the Ensemble website (http://ensemblgenomes.org). The screening conditions were |FC| > 2.0, *P* < .05. After data processing, 200 DELs were obtained: 74 DELs were upregulated, 136 DELs were downregulated (Fig. 2A), 984 DEGs, 567 DEGs were upregulated, and 417 DEGs were downregulated (Fig. 2B).

### 3.2. Screening of DSMs

**Figure 3.**
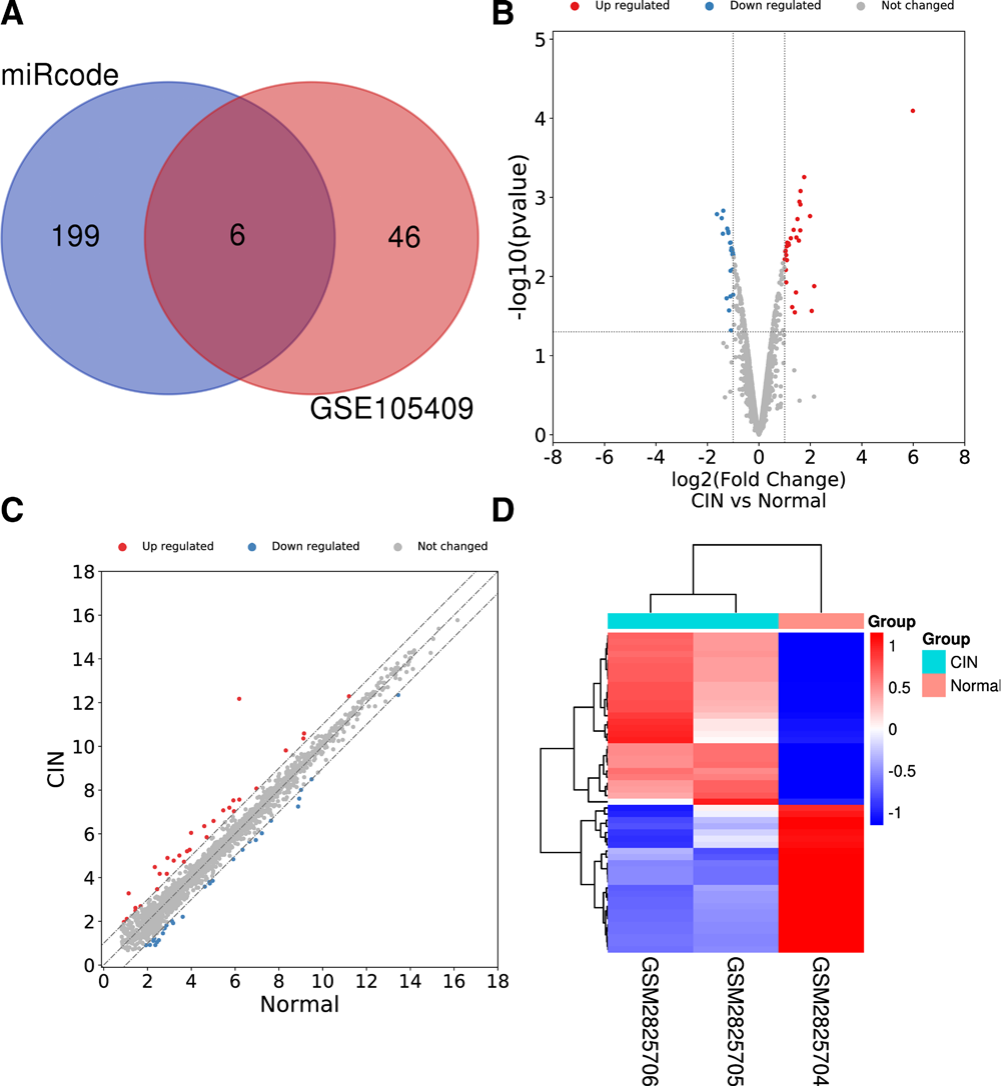
Identification of significant expression changes in RNAs in CIN. (A) miRcode and GSE105409 co-express differential miRNAs. (B) Volcano plots, (C) scatter plot and (D) heatmap with red for up-regulation and dark blue for down-regulation for miRNAs in GSE105409.

The GSE105409 dataset consists of five parts. GSM2825704 consisted of 16 normal patient samples, GSM2825705 consisted of 11 CIN I patient samples, GSM2825706 consisted of 29 CIN III patient samples, and the remaining two subsets were cervical cancer patient samples. The data was analyzed, and the screening conditions were the same as those in Section 2.1. A total of 52 DEMs were identified, of which 28 were upregulated and 24 were downregulated (Fig. 3A). The miRNAs corresponding to 200 DELs were predicted and analyzed using the database, and the co-expression differential miRNAs were selected from the prediction results and GSE105409 analysis results, and six DEMs (hsa-miR-142-3p, hsa-miR-10a-5p, hsa-miR-429 were upregulated and hsa-miR-139-5p, hsa-miR-338-3p, and hsa-miR-140-5p were downregulated) were screened (Fig. 3B).

### 3.3. DEGs screening and PPI network construction

**Figure 4.**
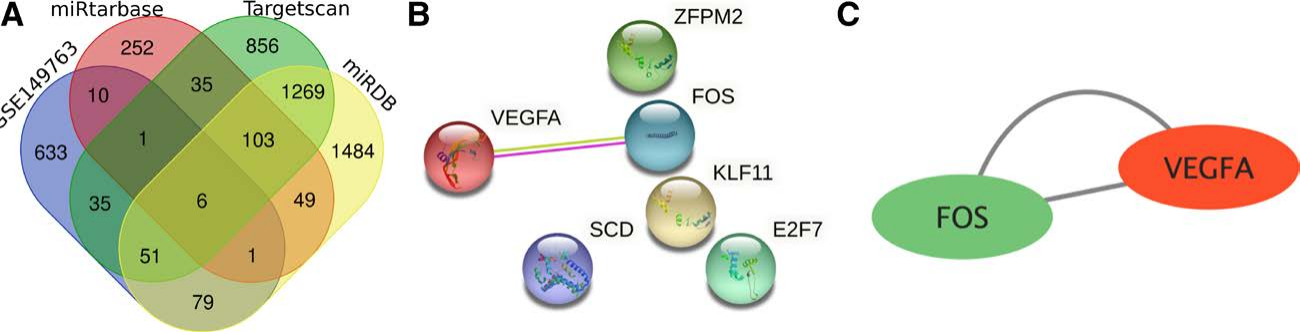
(A) The mRNA co-expression Venn diagrams of the GSE149764 dataset DEGs and DEMs were predicted by miRtarbase, Targetscan, and miRDB databases. (B) PPI network of DEGs. (C) For the screened core genes, red represents up-regulation and green represents down-regulation.

The target genes corresponding to the six DEMs obtained from the screening were predicted using the relevant database (Fig. 4A),^[16]^ and the predicted results were analyzed with the DEGs screened in 2.1 to take the intersection, and we obtained a total of six DEGs. DEGs were subjected to PPI network construction and core gene screening (Fig. 4B). The core genes were VEGFA and FOS (Fig. 4C).

### 3.4. Gene enrichment and KEGG analysis

**Figure 5.**
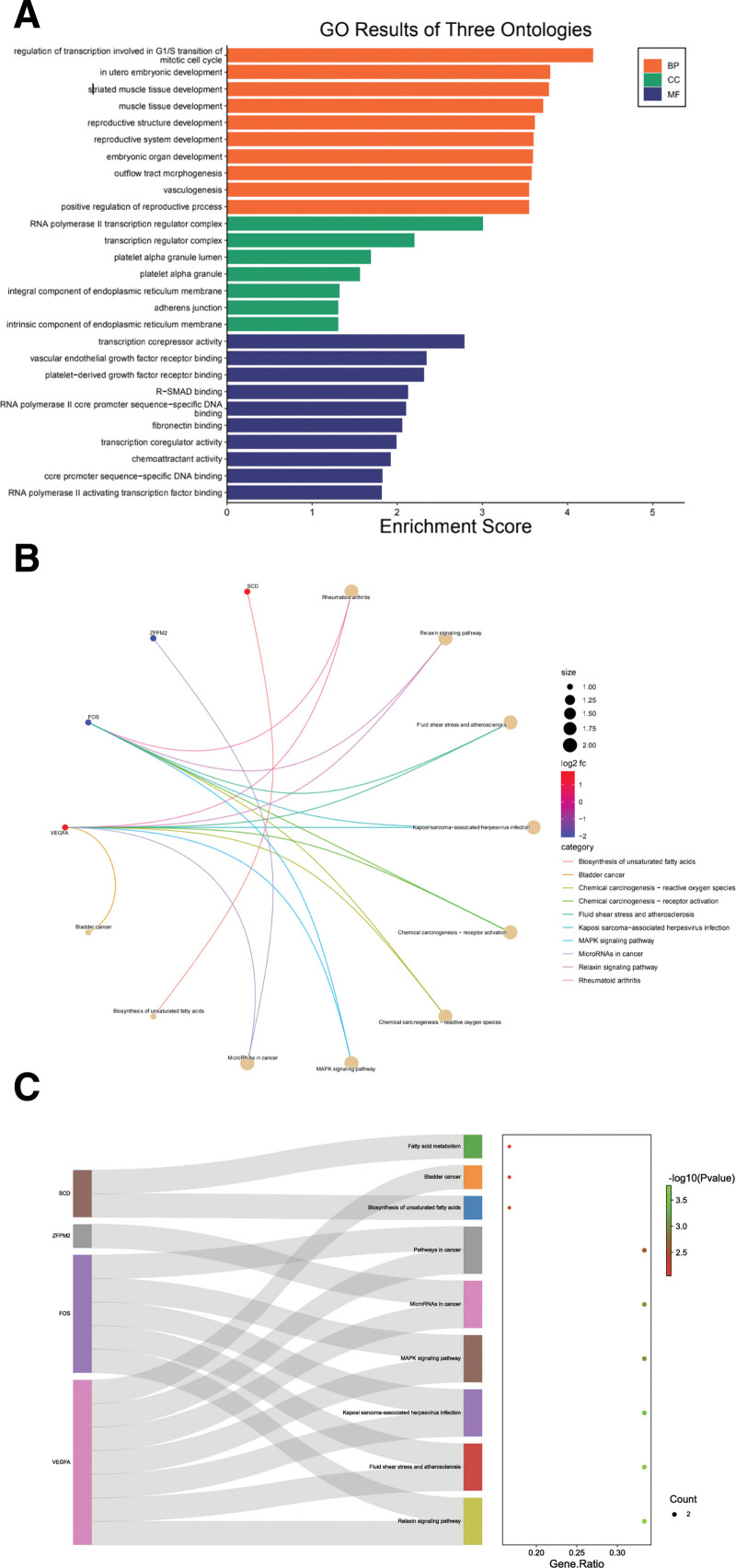
(A) GO function analysis of differentially expressed genes, (B) KEGG pathway analysis of DEG, (C) KEGG chord diagram expression profile, (D) The mechanism of action of the significant pathway and the position of DEG in the pathway.

The results of gene ontology (GO) analysis showed that the BP involved were mainly transcriptional regulation and angiogenesis in the G1/S transition of the mitotic cell cycle, and the molecular functions were the composition of RNA polymerase II transcriptional regulation complex, transcriptional regulation complex, and endoplasmic reticulum membrane. Partial and intrinsic components, adhesion junctions, cellular localization as transcriptional corepressor activity, vascular endothelial growth factor receptor binding, platelet-derived growth factor receptor binding, R-SMAD binding, RNA polymerase II core promoter sequence-specific DNA binding, fibronectin binding, etc (Fig. 5A). The KEGG pathway screening results showed significant differences in signaling pathways, such as the relaxin signaling pathway, chemical carcinogenesis-receptor activation, chemical carcinogenesis-reactive oxygen species, MAPK signaling pathway, and miRNA formation in cancer (Fig. 5B and C and Table 1). It can be seen from the key signaling pathways that the expression of VEGFA was increased and that of FOS was decreased, which may affect the occurrence and development of CIN from the perspective of cell differentiation (Fig. 5D).

**Table 1:** Results of the top 10 or total different terms and the top 10 KEGG pathway.

Category	ID	Description	*P* value	Gene count
BP	GO:0000083	Regulation of transcription involved in G1/S transition of mitotic cell cycle	.0000	2
BP	GO:0001701	In utero embryonic development	.0002	3
BP	GO:0014706	Striated muscle tissue development	.0002	3
BP	GO:0060537	Muscle tissue development	.0002	3
BP	GO:0048608	Reproductive structure development	.0002	3
BP	GO:0061458	Reproductive system development	.0003	3
BP	GO:0048568	Embryonic organ development	.0003	3
BP	GO:0003151	Outflow tract morphogenesis	.0003	2
BP	GO:0001570	Vasculogenesis	.0003	2
BP	GO:2000243	Positive regulation of reproductive process	.0003	2
CC	GO:0090575	RNA polymerase II transcription regulator complex	.0010	2
CC	GO:0005667	Transcription regulator complex	.0063	2
CC	GO:0031093	Platelet alpha granule lumen	.0204	1
CC	GO:0031091	Platelet alpha granule	.0276	1
CC	GO:0030176	Integral component of endoplasmic reticulum membrane	.0475	1
CC	GO:0005912	Adherens junction	.0499	1
CC	GO:0031227	Intrinsic component of endoplasmic reticulum membrane	.0499	1
MF	GO:0003714	Transcription corepressor activity	.0016	2
MF	GO:0005172	Vascular endothelial growth factor receptor binding	.0046	1
MF	GO:0005161	Platelet-derived growth factor receptor binding	.0049	1
MF	GO:0070412	R-SMAD binding	.0075	1
MF	GO:0000979	RNA polymerase II core promoter sequence-specific DNA binding	.0078	1
MF	GO:0001968	Fibronectin binding	.0088	1
MF	GO:0003712	Transcription coregulator activity	.0103	2
MF	GO:0042056	Chemoattractant activity	.0120	1
MF	GO:0001046	Core promoter sequence-specific DNA binding	.0149	1
MF	GO:0001102	RNA polymerase II activating transcription factor binding	.0153	1
KEGG	hsa05323	Rheumatoid arthritis	.0008	2
KEGG	hsa04926	Relaxin signaling pathway	.0015	2
KEGG	hsa05418	Fluid shear stress and atherosclerosis	.0017	2
KEGG	hsa05167	Kaposi sarcoma-associated herpesvirus infection	.0033	2
KEGG	hsa05207	Chemical carcinogenesis – receptor activation	.0039	2
KEGG	hsa05208	Chemical carcinogenesis – reactive oxygen species	.0043	2
KEGG	hsa04010	MAPK signaling pathway	.0075	2
KEGG	hsa05206	MicroRNAs in cancer	.0083	2
KEGG	hsa01040	Biosynthesis of unsaturated fatty acids	.0132	1
KEGG	hsa05219	Bladder cancer	.0201	1

BP = biological processes, CC = cellular components, GO = gene ontology, KEGG = Kyoto Encyclopedia of Genes and Genomes.

### 3.5. ceRNA network construction

**Figure 6.**
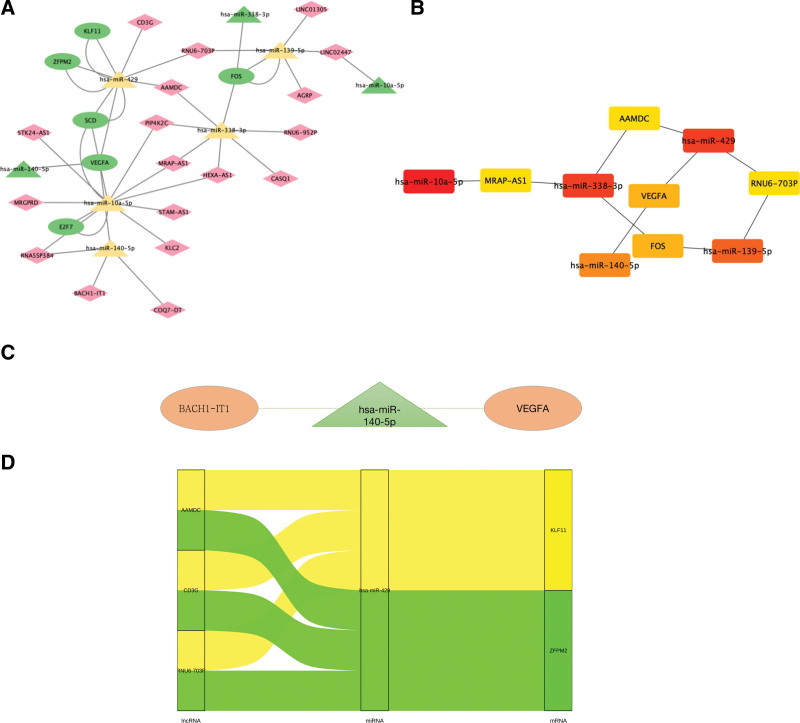
(A) ceRNA network construction. This network contains 18 lncRNAs, 5 miRNAs, and 6 mRNAs, with a total of 29 connection points and 36 edges. V represents lncRNA, rectangle represents miRNA, and oval represents mRNA. (B) Calculate the top 12 targets using the Cytohubba plugin. (C) One ceRNA networks in compound conditions, green represents down-regulation and orange represents up-regulation. (D) A multi-pathway connection network of miR-429.

Upload DELs, DEMs, and DEGs to Cytoscape 3.9.0 software to draw a ceRNA network diagram and establish an interaction diagram with miRNA as the midpoint (Fig. 6A). The network construction included 18 DELs, five DEMs, and six DEGs. Topology analysis revealed eligible interacting networks (Fig. 6B). Based on the competitive binding of miRNAs to lncRNAs and mRNAs, two eligible ceRNA pathways were selected (Fig. 6C and D): upregulated miRNA expression and downregulated lncRNA and mRNA expression, or downregulated miRNA expression and upregulated lncRNA and mRNA expression (Table 2).

**Table 2:** ceRNA network elements and their relationships.

Category	Name	FC	*P* value	Ligation target	Regulation
lncRNA	AGRP	–11.99142626	.003842623	hsa-miR-139-5p	Down
lncRNA	LINC02447	–57.19903528	.005694711	hsa-miR-139-5p, hsa-miR-10a-5p	Down
lncRNA	RNU6-703P	–103.3792108	2.53028E–06	hsa-miR-139-5p, hsa-miR-429	Down
lncRNA	RNU6-952P	–62.15052008	.015342047	hsa-miR-338-3p	Down
lncRNA	AAMDC	–11.39779768	.011153127	hsa-miR-338-3p, hsa-miR-429	Down
lncRNA	PIP4K2C	–18.65667399	.007930616	hsa-miR-338-3p, hsa-miR-10a-5p	Down
lncRNA	HEXA-AS1	–14.15957657	.03344961	hsa-miR-338-3p, hsa-miR-10a-5p	Down
lncRNA	CASQ1	–7.83888897	.049716249	hsa-miR-338-3p	Down
lncRNA	KLC2	–26.32732282	.003439861	hsa-miR-10a-5p	Down
lncRNA	MRGPRD	–63.04270267	.008761779	hsa-miR-10a-5p	Down
lncRNA	RNA5SP384	–71.1015127	.004491879	hsa-miR-429	Down
lncRNA	CD3G	–47.17766148	.016652371	hsa-miR-140-5p	Down
lncRNA	COQ7-DT	–22.70934854	.040570617	hsa-miR-10a-5p, hsa-miR-140-5p	Down
lncRNA	LINC01305	16.30439224	.015324906	hsa-miR-139-5p	Up
lncRNA	MRAP-AS1	24.69801458	.010367306	hsa-miR-338-3p, hsa-miR-10a-5p	Up
lncRNA	STK24-AS1	21.2820856	.03051205	hsa-miR-10a-5p	Up
lncRNA	STAM-AS1	34.19650262	.032609663	hsa-miR-10a-5p	Up
lncRNA	BACH1-IT1	5.085034058	.015407514	hsa-miR-140-5P	Up
mRNA	FOS	–1.909190607	.528860977	hsa-miR-139-5p, hsa-miR-338-3p	Down
mRNA	ZFPM2	–2.05333474	.302003296	hsa-miR-429	Down
mRNA	KLF11	–1.059772788	.386314096	hsa-miR-429	Down
mRNA	VEGFA	1.749509	.296080442	hsa-miR-429, hsa-miR-140-5p	Up
mRNA	E2F7	2.556256281	.262402236	hsa-miR-10a-5p	Up
mRNA	SCD	1.764538913	.403837061	hsa-miR-10a-5p, hsa-miR-429	Up
Category	Name	logFC	*P* value	Ligation target	Regulation
miRNA	hsa-miR-139-5p	–1.0211893	.008163	FOS	Down
miRNA	hsa-miR-338-3p	1.00248	.00248	FOS	Down
miRNA	hsa-miR-140-5p	–1.0795601	.0046545	VEGFA	Down
miRNA	hsa-miR-10a-5p	2.1488868	.0132375	E2F7, SCD	Up
miRNA	hsa-miR-429	1.0583154	.0118604	SCD, ZFPM2, KLF11	Up

lncRNAs = long non-coding RNAs, miRNAs = microRNAs.

### 3.6. Key gene expression and prognosis analysis

**Figure 7.**
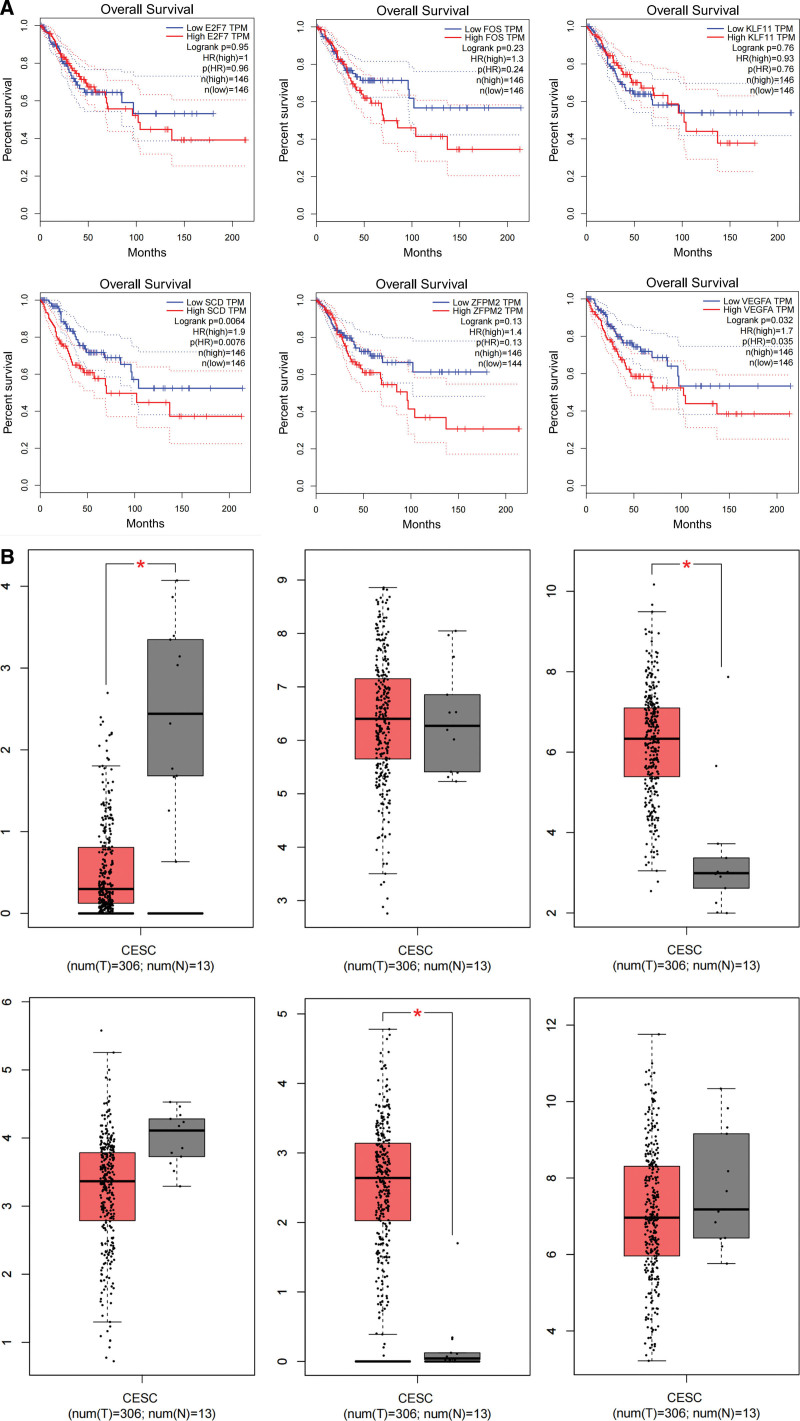
(A) The expression levels of key genes in different samples. The expression of E2F7, SCD, and ZFPM2 was significantly different between normal and cervical cancer samples. (B) Prognosis of key genes. The results of the survival analysis showed that the expression levels of VEGFA and SCD were related to the overall survival of the patients (*P* < .05).

The relationship between cervical squamous intraepithelial lesions and cervical cancer is similar. Therefore, using cervical cancer as an example, we predicted the prognostic impact of key genes in cervical cancer and cervical squamous intraepithelial lesions (Fig. 7A and B).

## 4. Discussion

Cervical squamous intraepithelial lesions are currently known to be closely related to lesions in cervical cancer. Known as a more common tumor disease in women, the incidence of cervical cancer is increasing in China, and cervical squamous intraepithelial lesion is a precancerous process of the cervix, which is closely related to the occurrence of invasive cervical cancer,^[18]^ Although part of the LSIL can regress on its own under the action of the body’s immune function, the persistence of HSIL increases the risk of cervical cancer. Therefore, we actively treat high-grade cervical lesions to effectively reverse cervical squamous intraepithelial lesions, block the progressive development of the disease, and reduce the incidence of cervical cancer.^[19]^ The existing treatment methods for HISL are cervical conization, annular electrosurgical excision (LEEP knife), and cryotherapy. Although surgery can remove the lesions, it has no therapeutic effect on persistent viral infection and it can easily cause the second trimester of pregnancy in pregnant women. Miscarriages and premature births are prone to minor or massive hemorrhage, infection, minor damage to related organs, and residual lesions after surgery. There is a certain recurrence rate in long-term observations. Therefore, a noninvasive and reliable treatment method is sought to fundamentally remove it^[20]^ which is of great significance in preventing and treating recurrence.

**Figure 8.**
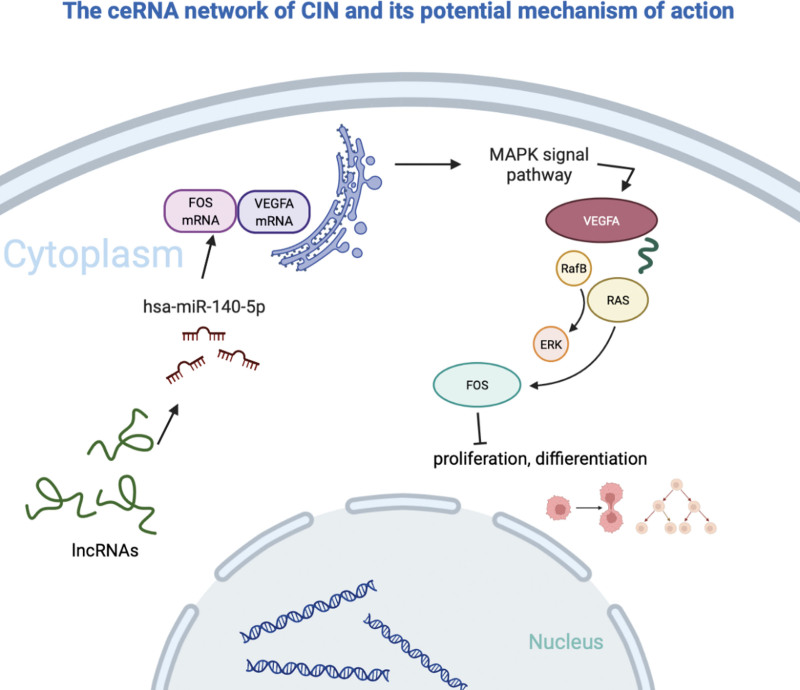
Using online prediction analysis of RNA sequence (http://www.csbio.sjtu.edu.cn/bioinf/lncLocator/), we found that lncRNA, miRNA and mRNA are all located in the cytoplasm. Regarding the ceRNAs network of CIN and its potential mechanism of action, the dotted box is the part of the later experimental verification.

The mechanism of action of ceRNAs involves linking mRNA with numerous non-coding RNAs to promote the degradation of the target or inhibit its translation by competing for shared transcripts that miRNAs mutually regulate at the post-transcriptional level. The activity of ceRNAs may be related to their abundance, mutual affinity, and subcellular localization.^[21]^ Studies have shown that the lncRNA PTENP1 can upregulate PTEN expression by competitively binding to miR-106b and inhibiting the growth of cervical cancer cells by promoting apoptosis, inhibiting proliferation, and epithelial-mesenchymal transition.^[22]^ In addition, the non-coding RNA circEPSTI1-miR The -375/409-3P/515-5p-SLC7A11 axis can affect cervical cancer proliferation in a competitive binding manner, suggesting that non-coding RNAs can serve as new useful biomarkers for monitoring and therapeutic targets in cervical cancer. However, most of these studies have focused on cervical cancer, and research on the molecular mechanism of cervical precancerous lesions is insufficient. This study aimed to clarify the possible mechanism of CIN or cervical precancerous lesions by constructing ceRNAs for further experimental and clinical research (Fig. 8). Provide some reference. Using bioinformatics technology, the significant differences between the lncRNAs BACH1-IT1/miR-140-5p/VEGFA and the MAPK signaling pathway in cervical precancerous lesions were identified and a multi-pathway connection network of miR-429 was constructed. We chose the BACH1-IT1/miR-140-5p/VEGFA signaling axis instead of the multi-pathway connection network of miR-429 because the subsequent key genes of the multi-pathway connection network of miR-429 did not form a study pathway. miR-140-5p shows low expression in a variety of tumor diseases, and affects tumor cell proliferation, migration, cell cycle, differentiation, etc,^[23]^ and it can be regarded as a potential tumor suppressor gene. VEGFA also improved microvascular permeability. Promote endothelial cell proliferation, vascular construction and migration from different tissues,^[24]^ studies have shown that miR-140-5p can inhibit the proliferation, migration and invasion of lung and colorectal cancer cells by targeting VEGFA.^[25]^ In a study on osteosarcoma, overexpression of VEGFA promoted cell viability, invasion, and migration and inhibited apoptosis, whereas overexpression of miR-140-5p partially inhibited apoptosis.^[26]^ The Fos are regarded as proto-oncogenes and include c-Fos (the human homolog of the retroviral oncogene v-Fos), FosB, etc, which together with members of the Jun family (c-Jun, JunB, and JunD) form the AP-1 proteome. The protein is a target gene after dimerization with promoter and enhancer regions, and the transition of Fos gene expression is an important step in carcinogenesis and/or progression^[27]^ affecting cancer cell survival and metabolism. In early cervical precancerous lesions, c-Fos expression is relatively low and overall AP -1 binding activity is low, whereas c-Fos expression is high in aggressive cervical cancer.^[28]^ Activation of the MAPK signaling pathway can act on downstream targets to regulate gene expression and affect cell proliferation, migration, apoptosis,^[29]^ epithelial-mesenchymal transition, and treatment.

## 5. Conclusions

In vitro experimental studies have found that MAPK/ERK activation of the PI3K/AKT signaling pathway can regulate VEGF family factors and affect the expression of cervical oncogenes, which may contribute to the development of cervical cancer.^[30]^ The BACH1-IT1/miR-140-5p/VEGFA pathway axis, MAPK signaling pathway, and key factors VEGFA and FOS may be potential therapeutic targets in cervical intraepithelial neoplasia. This discovery has molecular implications for the future prevention and treatment of cervical diseases.

## Acknowledgments

We acknowledge Dr Yiming SUN, who helped improve the scientific quality of this study.

## Author contributions

**Data curation:** Buwei Han, Ning Liu, Rui Zheng.

**Formal analysis:** Ding Qi, Shimeng Wang, Shuoqi Wang.

**Funding acquistion:** Hongmei Li, Li Liu.

**Supervision:** Ding Qi, Li Liu.

**Writing – original draft:** Ding Qi.

**Writing – review &amp; editing:** Ding Qi, Li Liu.
